# Supplementary material for: Are carbon emissions trading and green financial instruments synergistic? -Comprehensive quantitative research based on content analysis
Source: PLoS One. 2024 Mar 7;19(3):e0298601. doi: 10.1371/journal.pone.0298601 (PMC10919601; doi:10.1371/journal.pone.0298601)
Supplement: S1 Data — (ZIP) [file pone.0298601.s001.zip › Data/地级市Z_F工作报告.zip!/╔┬╬≈/▒a╝a╩╨/└┤╫╘╣1⁄2╓┌║┼í╛┬φ┐╦╩2╛▌═°í┐_2010.docx]

各位代表：

　　现在，我代表市人民政府向大会报告工作，请予审议，并请市政协委员和其他列席人员提出意见。

2009年工作回顾

　　过去的一年，在省委、省政府和市委的坚强领导下，市政府认真学习实践科学发展观，全力以赴保增长，改善民生促和谐，圆满完成了市十三届人大三次会议确定的各项任务。全年实现地方生产总值806.6亿元，同比增长15%；完成财税总收入91.8亿元，增长19.2%；地方财政收入30.1亿元，增长26%；城镇居民人均可支配收入16346元，增长23.6%；农民人均纯收入4186元，增长19.6%。一年来，面对金融危机带来的严重冲击，我们采取了一系列保增长的政策措施，经济增速位居全省前列；抢抓国家扩大内需的历史机遇，超前谋划实施了一批重大基础设施和产业项目，突破了长期以来制约宝鸡发展的瓶颈；顺应人民群众对创建最佳宜居城市的迫切愿望，扎实开展创建活动，被授予国家森林城市、国家节水型城市、全国环境优美城市称号，荣获中国人居环境奖，跻身“新中国60个城市发展代表”行列，宝鸡的发展站在了新的历史起点上。

　　保增长成效显著，工业发展全面提速。把抓工业作为保增长的重点，全力以赴帮助企业渡过难关，工业经济逆势上扬。全市规模以上工业企业净增96户，完成增加值342.4亿元，增长19.8%，增速持续位居全省第一。大企业大集团发展势头迅猛，12户大企业大集团完成总产值486亿元，占规模以上工业的49%，东岭集团销售收入突破200亿元，宝石钢管跨入百亿企业行列。国企改革迈出新步伐，宝鸡机床与秦川机床合资、长岭股份破产重整及6户政策性破产企业重组工作顺利完成。高新区实现生产总值240亿元，增长25.5%。技术创新取得新成绩，新建成3个省级工程技术研究中心，荣获全国科技进步考核先进市。节能减排成效明显，万元生产总值能耗降低5%。

　　项目建设扎实推进，发展基础更加坚实。全年实施重点项目100个，完成投资410亿元，项目投资额、大项目数、竣工投产项目数均比上年翻番。法士特消失模铸造、石油钢管连续油管生产线、大唐宝鸡热电等项目建成投用，宝二电扩建等项目建设进展顺利。全年完成全社会固定资产投资639.1亿元，增长41.7%。围绕落实关天规划和产业振兴规划，高起点谋划储备了260个重大战略项目，总投资1485亿元。争取中省各类资金86.3亿元，增长34.7%。全年合同引资637.9亿元，到位资金247.6亿元，招商引资工作受到省政府表彰。

　　文化旅游取得突破，第三产业蓬勃发展。成功举办了第二十届西交会和“两节两会”，法门寺合十舍利塔、中华石鼓园建成对外开放，中华礼乐城等十大工程建设全面启动。周秦文化产业示范区规划通过国家级评审，成为全省重点支持的文化园区。全年共接待游客1297万人次，实现旅游综合收入78亿元，增长21%。金融业快速发展，全年新增贷款106亿元，是上年增量的两倍多，浦发银行、交通银行对外营业，凤翔县荣获全国金融生态县。商贸物流业加速发展，陈仓物流园区会展中心、开元商城、名汇购物广场建成投用，住房、汽车、家电等消费持续升温。全年实现社会消费品零售总额280.1亿元，增长19.5%。第三产业持续快速增长，实现增加值230.3亿元，增长13.9%。

　　县域经济实力壮大，农民收入大幅提高。农业产业化程度进一步提高，市级以上龙头企业达到136户。现代牧业公司两万头奶牛场、7个千头奶牛场、48个标准化奶牛小区、5万头良种仔猪繁育场建成投用，奶牛和生猪存栏分别达到17.7万头和 98.6万头，果品总产量77.4万吨，粮食总产达到165.6万吨，形成了畜为主导、果为特色、粮为基础的发展格局。县域工业化取得新进展，安排 5000万元支持重点园区建设，9个省级重点县域工业园新开工项目53个，县域经济、非公经济在全市经济中的比重分别达到45.2%、47.6%。凤翔、扶风县跻身全国县域经济竞争力提升最快百强县。城镇化步伐显著加快，启动了关中百镇 21个项目和 12个省级重点镇建设，一批道路、水电等基础设施工程陆续开工。新农村建设扎实推进，新修改建农村公路1000公里、饮水工程243处、沼气池2.1万口，全市五星村、四星村分别达到100个和300个。

　　大城市建设步伐加快，载体服务功能明显提升。编制完成了新一轮城市总体规划和土地利用规划，城市空间布局进一步优化。宝天高速建成通车，西宝高速改扩建、宝平高速、西宝快速铁路客运专线开工建设，凤凰、蔡阳、龚刘渭河大桥和陈仓大道东段、宝鸡高速客运中心等项目建成投用，城市交通体系日趋完善。5万吨中水回用、冯家山引水复线、老城区重点区域集中供热改造等工程顺利完工，公共服务功能进一步增强。国家卫生城市顺利通过复检，县县建成了污水处理厂、垃圾处理场，城市环境质量连续3次位列全省第一。生态文明家园建设取得新成效，陇县等11个县区通过国家生态示范县验收，凤翔、麟游、千阳县创建成为国家园林县城，凤县荣获全国最美小城殊荣。数字化城管系统延伸至陈仓区，我市被列为全国数字化城市管理试点城市。

　　社会事业全面发展，民生得到持续改善。启动实施了2309个民生项目，完成投资45.4亿元，比上年增加13亿元，投资额为历年之最。新型农村社会养老保险全面推行，全市60岁以上老人喜领养老金。率先在全省建立了被征地农民就业和社会保障制度，城镇居民养老保险、医疗保险和廉租房保障工作扎实推进，在制度层面上实现了无缝隙、全覆盖。中小学布局调整步伐加快，高考质量进一步提升，渭滨区建成省级“教育强区”。医药卫生体制改革进展顺利，基层医疗卫生服务体系进一步健全，麟游县荣获全国农村中医工作先进县。市直文化单位体制改革启动实施，8个项目入选省第二批非物质文化遗产名录。竞技体育和群众体育蓬勃开展，成功举办了全国竞走大奖赛、省第六届残疾人运动会。“十万贫困户增收行动计划”顺利实施，突破西山成效明显，全市10.5万人实现温饱和脱贫，22.4万人饮水安全问题得到解决。农村灾民住房重建全部完成，累计完成灾后重建投资48亿元。市流浪未成年人救助中心、儿童福利院项目基本完工，3个县级中心敬老院、3个县级社区服务中心建成投用，渭滨、金台区荣获全国和谐社区示范城区。人口计生工作质量进一步提高，金台区荣获国家级计划生育优质服务先进区。国土资源管理进一步加强，为经济社会发展提供了有力保障。防震减灾能力日益增强，地震前兆信息和技术服务系统建成投用。实施了寄宿制中小学蛋奶工程、农村育龄妇女健康检查治疗、供热供气扩容、宝虢凤收费站撤站等十件惠民实事，人民群众得到更多实惠。

　　人事、监察、审计、统计、广电、人防、气象、档案、地志、双拥、民族宗教、国家安全、妇女儿童、侨务外事、扶残助残、慈善事业等各项工作取得新成绩。

　　一年来，我们不断加强民主法制建设和政府自身建设，自觉接受市人大、市政协的监督，广泛听取人大代表、政协委员、民主党派以及专家学者的建议和意见，认真办理人大代表建议、意见和政协委员提案。深入开展学习实践科学发展观活动，在政府系统全面推行干部学习教育、重大事项科学决策、畅通民意接受监督、政府大督查四项工作制度，完善新闻发布机制，开通了市政府领导和28个部门领导信箱，机关作风进一步转变，办事效率和为民服务水平显著提高。应急管理机制不断完善，处置公共突发事件的能力进一步增强。严格落实安全生产责任制，安全生产形势总体平稳。广泛开展“五五”普法，深入排查调处矛盾纠纷，信访突出问题正在逐步解决。平安宝鸡创建深入推进，广大群众对社会治安满意率达到92.4%，位居全省前列。

　　各位代表，过去一年，在金融危机的严重影响下，我们取得这样令人鼓舞的成绩，这是市委科学决策、正确领导的结果，是市人大、市政协及社会各界大力支持、共谋发展的结果，是全市广大干部群众同心同德、顽强拼搏的结果。在此，我代表市政府，向给予我们支持和监督的人大代表、政协委员、各民主党派、工商联、各人民团体、社会各界人士，向驻宝单位、解放军和武警驻宝部队，表示崇高的敬意和衷心的感谢！

　　在充分肯定成绩的同时，我们也要正视发展中面临的困难和问题。主要是：经济总量不大，大企业数量不多，大产业没有真正形成；经济结构仍不尽合理，县域经济实力依然不强，非公经济发展缓慢，现代服务业相对滞后；农村基础设施建设薄弱，农民持续增收困难，统筹城乡发展压力较大；城镇化水平不高，辐射带动能力不强，城市重大基础设施还不能支撑特大城市建设的需要；一些干部创新意识不强，思想解放不够，工作作风不实，发展环境需要进一步优化。对这些问题，我们必须高度重视，采取有力措施，认真加以解决。

2010年政府工作

　　今年，是全面实施《关中—天水经济区发展规划》的开局之年，更是我市巩固经济回升向好趋势、实现率先发展的重要一年。当前，已进入后国际金融危机时期，世界经济全面复苏的不确定因素依然较多。国内经济回升的基础还不牢固，经济增长的内在动力仍显不足，经济发展的外部形势依然严峻。但是，我们也应看到，今年的经济发展环境将好于去年。国家继续实施积极的财政政策和适度宽松的货币政策，出台了发展新兴产业、加快城镇化步伐、扩大消费等一系列重大措施；颁布的关天规划将宝鸡的发展首次上升到国家战略，定位为关天经济区副中心城市，一批涉及宝鸡长远发展的重大项目列入了国家盘子；国家还将出台西部大开发新的十年规划，进一步加大对西部地区基础设施建设和优势产业的扶持力度，为我市发展提供了新的历史机遇。

　　纵观宝鸡发展，连续多年的较快增长，为今后一个时期加快发展积蓄了强大势能；大企业大集团建设取得明显成效，成为未来发展的重要支撑；谋划和实施的一批重大基础设施项目，增强了加快发展的承载能力；社会保障体系日益完善，创造了和谐稳定的社会环境；经过抗震救灾、应对金融危机等工作的锻炼，全市干部群众形成了加快发展的共识，焕发了奋发有为的精神状态，为宝鸡率先发展凝聚了强大的精神力量。这些因素，都为我市今年乃至今后一个时期的持续快速发展提供了有利条件。我们必须坚定信心，抢抓机遇，凝心聚力，积极作为，以攀高争先、敢闯一流的雄心壮志，努力开创宝鸡经济社会发展新局面！

　　今年政府工作的总体要求是：坚持以科学发展观为指导，认真贯彻落实党的十七届四中全会、中央经济工作会议和省市委全会精神，紧紧围绕“四个第一”、“五大突破”的思路目标，强力推进“率先发展八大工程”，以关天规划实施为契机，在更高层次上谋划未来发展；以转变经济发展方式为主线，加快培育壮大支柱产业；以重点项目建设为抓手，扩大固定资产投资规模；以统筹城乡发展为重点，全面提高“三农”发展水平；以建设生态园林特大城市为目标，提升城市辐射带动能力；以改善和保障民生为根本，促进和谐社会建设，奋力实现宝鸡发展的新跨越。

　　主要预期目标是：生产总值确保增长14%，力争15%；全社会固定资产投资增长30%；地方财政收入增长15%以上；规模以上工业增加值增长18%以上；城镇登记失业率控制在4.2%以内；万元GDP能耗下降4%以上；二氧化硫、化学需氧量分别削减4.3%和2.8%以上；农民人均纯收入和城镇居民人均可支配收入均增长15%以上；人口自然增长率控制在5.6‰以内。

重点抓好以下九个方面的工作：

　　（一）把握大势超前谋划，全面提高宝鸡科学发展水平

　　国际金融危机导致世界经济增长格局出现新的重大变化，促进经济结构调整，加快经济发展方式转变，已成为今后一个时期经济工作的主基调。我们将抢抓关天规划实施机遇，把加快推进“率先发展八大工程”，作为顺应国内外经济发展趋势、转变发展方式的重要举措，推动宝鸡科学发展、和谐发展。

　　加快推进关天规划实施。按照关天规划对宝鸡的战略定位，今后一个时期，我们要以“一个枢纽、三大基地、两个示范区”为支撑，全力建设生态园林特大城市和经济区副中心城市。“一个枢纽”，就是超前建设适应未来发展需要的立体交通网络，使宝鸡成为贯通东西、连接南北的西部交通枢纽；“三大基地”，就是先进装备制造业基地、新材料研发生产基地、姜炎周秦文化基地，重点建设以先进装备制造业为主导，配套能力强、产业集聚效应明显的工业强市，打造区域性文化旅游和商贸物流中心；“两个示范区”，就是统筹科技资源改革示范区、统筹城乡发展示范区，使科技创新能力和城乡一体化水平位居经济区前列。到2020年，全市的生产总值达到3500亿元，力争3800亿元，市区人口达到130万，建成区面积达到140平方公里，城镇化率达到60%，社会保障体系和城乡居民收入位居全省前列，社会事业全面进步，公共服务均等化水平不断提高。要按照这一目标，加快落实我市关天规划实施意见，抓紧编制县域发展规划和部门专项规划，高质量完成“十二五”规划编制工作。

　　加快推进经济发展方式转变。把我市的经济增长由单纯依靠投资拉动向依靠消费、投资、外贸协调拉动转变，由主要依靠第二产业带动向依靠第一、第二、第三产业协同拉动转变，由数量规模型向质量效益型转变。积极扶持县域经济发展，尽快改变“市强县弱”的现状，优化区域经济结构。落实支持非公经济发展的各项政策措施，提高非公经济比重，促进所有制结构改善，使非公经济成为我市吸纳就业、财税增收的重要力量。推进传统产业技术改造，发展战略性新兴产业，提升文化旅游等第三产业发展水平，促进宝鸡各类产业在更高层次上协同发展。以城镇化为突破口，积极实施城乡一体化发展战略，探索从根本上解决“三农”问题的途径。以技术创新为引领，提高企业核心竞争力，加快推进创新型城市建设。积极引导建立以低碳排放为特征的产业体系和消费模式，大力发展循环经济，增强可持续发展能力。增加中低收入者的收入，不断提高最低工资标准和社会保障标准，为扩大内需、拉动消费奠定基础。

　　加快推进“率先发展八大工程”。以“五大突破落实年”为主题，以项目建设为抓手，全力推进龙头企业带动、园区突进、农业农村“三增一创”、文化旅游精品、交通枢纽提升、城镇化推进、市场体系建设、生态环保等“率先发展八大工程”建设。进一步聚集整合各类要素资源，加大投入力度，引导更多的资金投向“八大工程”，并在规划、用地、项目审批等方面给予倾斜，优先保障，确保“八大工程”顺利实施，促进宝鸡在关天经济区中率先发展。

　　（二）强力推进集群化发展，壮大工业经济实力

　　按照“优化传统产业、提升优势产业、抢抓新兴产业”的思路，坚持加快发展与提质增效并举，扩张总量与优化结构并重，推动工业超常规发展。

　　以大企业带动形成大产业。围绕大企业大集团建设，突出抓好新扩技改项目、工业园区发展和中小企业配套，全力促进大企业大集团快速成长，形成产业集群，带动大产业发展。完善大企业培育体系，扶持30户高成长性重点企业提速发展，形成大企业群体。引导中小企业加强产业配套，延伸产业链条，壮大产业规模。重点培育有色金属冶炼、汽车及零部件、石油装备、机床工具、能源化工等几个千亿元级和五百亿元级的工业支柱产业，形成工业依靠支柱产业拉动、支柱产业由骨干企业支撑、骨干企业靠知名品牌提升的新格局。年内力争陕汽集团（宝鸡部分）、宝钛集团产值过百亿。

　　大力发展新兴产业。围绕总投资110亿元的15个新能源产业项目规划，集中抓好太阳能光伏、风电装备及关键零部件制造、节能设备三大重点产业。以建设国家新材料高技术产业基地为契机，加速推进国核锆管、核电材料制造、中国钛交易中心等重点项目，打造“中国钛谷”。以宝成航空仪表、长岭电子科技、凌云电器、烽火通信为主体，加快建设航空安全装备产业园。大力发展环保新技术产业，支持海浪锅炉等高效、节能、低碳产业扩大规模，提高市场占有率。

　　加快技术创新步伐。围绕提高科技创新能力，加快建设宝鸡科技资源中心和科技投资公司。支持建设钛及钛合金材料、石油钻采装备、焊接钢管三个国家级工程技术中心，创建节能环保锅炉、高可靠性无线通信、飞行环境监测三个省级工程技术中心。实施“高层次人才集聚计划”，面向国内外吸引高层次人才，培养一批高级技能型人才。支持一批产学研项目，促进企业与科研院所、大专院校合作，让更多科技创新项目在宝鸡落地生根。以国家知识产权示范城市创建为契机，加强知识产权的创造、运用和保护。推动工业化与信息化融合发展，改造提升烟酒、食品、纺织、建材等产业的整体水平。

　　切实抓好节能减排。积极推进节能减排减碳，加大企业技术改造力度，杜绝新上高耗能、高污染项目，加快淘汰落后生产能力，突出抓好水泥、化工、冶炼、造纸等重点领域和重点企业的节能改造和污染治理。大力开展机关、公共机构节能活动，加快推广节能新技术、新工艺、新产品。加大节水型社会建设力度，完善节水政策措施，改造城镇供水管网，普及工农业节水技术，节约生产生活用水。

　　（三）扎实做好“三农”工作，推进城乡统筹发展

认真贯彻中央农村工作会议精神，以农民增收为核心，以统筹城乡为突破口，进一步加大“三农”投入力度，壮大特色优势产业，提升新农村建设水平，加快城乡一体化发展步伐。

　　大力发展现代农业。稳定粮食种植面积，推广优质专用品种和先进技术，提高单产和品质，推进增产增效。加快百万亩苹果、百万亩花椒、百万亩核桃、百万亩蔬菜和60万亩猕猴桃基地建设，实施10万头高产奶牛养殖基地建设工程，加快发展各类畜牧养殖小区，支持千阳县全省奶牛标准化养殖小区示范县建设。深化与杨凌的农业科技合作，提高农业科技专家大院水平，抓好农业科技示范园区建设，打造现代农业高技术产业基地。推进农业产业化经营，扶持秦宝牧业、蒙牛乳业、华龙牧业、现代牧业等企业开展基地建设，打造品牌，开拓市场。引进一批国内知名饲料、辣椒、蔬菜等加工企业，壮大农业产业化龙头企业群体。大力支持农产品流通体系建设，在市区建设大型农产品物流中心，在每个县区建设农产品综合批发市场，在乡镇和重点村建设专业市场，形成三级农产品流通服务网络。大力发展农民专业合作社，使每一个产业都有龙头企业带动、合作组织联接、科技园区支撑，引领现代农业发展。

　　千方百计增加农民收入。加强农村实用技术和农民工技能培训，促进农村劳动力转移，拓展非农就业增收空间。以“举措到户”工程为抓手，加快实施促进农民增收的8个专项规划，大力发展一村一品、一县一业，挖掘农业内部增收潜力。积极探索农村产权确权和交易办法，促进农村产权“依法、自愿、有偿”流转，使农民获得更多的财产性收入。全面落实各项涉农补贴，不断增加农民转移性收入。

　　加快改善农村基础设施条件。实施农业综合开发、农村饮水安全、病险水库除险加固、中小河流治理等工程，发展节水灌溉10万亩，解决15万人饮水困难。大力实施中低产田改造和土地复垦整理，抓好高标准农田建设，提高农业综合生产能力。继续实施突破西山战略，启动60个扶贫重点村建设，完成8万农村贫困人口脱贫任务。新改建县乡公路260公里，新建通村公路500公里，解决最后20个行政村不通油路或水泥路问题，实现90%以上的行政村通达客运班车。推进新农村晋星创建，年内使五星村、四星村分别达到130个和370个。抓好农村环境综合整治，加大乡村垃圾清运处理力度，不断改善农村环境面貌。

　　积极推进城乡统筹。城乡统筹发展，是今后一个时期从根本上解决三农问题、扩大内需、转变发展方式、全面实现小康目标的战略选择。要以统筹城乡规划、产业发展、基础设施建设和公共服务为重点，促进公共资源、生产要素均衡配置和自由流动，建立以工促农、以城带乡的长效机制。抓住省上支持关中百镇和重点镇建设的有利机遇，在县城和重点镇组建城投公司，拓宽融资渠道，完善基础设施体系，加快形成以9个县城和12个重点镇为支撑的城镇群，提高县域城镇化水平。在蔡家坡和高新区开展统筹城乡一体化试点,使其很快成为全市城乡一体化发展的先行区。加快凤县全省统筹城乡改革试验，支持其在城乡一体化方面率先突破。放宽农民进城落户政策，加速农村劳动力向城镇转移，促进城乡共同繁荣。

　　（四）加大重点项目建设力度，集聚发展后劲

坚持把项目建设作为增强实力、累积优势的核心举措，以更开放的姿态，更长远的眼光，一手抓紧现有项目的实施，一手抓好长远项目的谋划。

　　全力抓好重点项目建设。今年，全市安排重点建设项目100个，总投资1508亿元，年度投资500亿元，力争竣工项目35个，带动全社会固定资产投资达到830亿元。突出抓好重大产业项目，加快宝石钢管工业园、秦川铸造工业园、宝石机械异地扩建、西凤酒扩建等项目建设进度，早日开工建设宝鸡风电产业园、宝成航空精密制造、数控机床研发生产基地等项目，年内完成宝二电二期扩建和千阳海螺、凤翔冀东水泥生产线等项目。切实做好项目保障服务工作，整合挖潜存量土地，鼓励和扩大民间投资，加强协调服务，确保重点项目顺利实施。

　　超前谋划一批重大项目。紧跟国家产业政策和投资方向，围绕特大城市建设、工业结构调整、文化旅游和商贸物流等方面，谋划一批事关宝鸡长远发展的重大项目。继续加大争取力度，确保项目总量增加、质量提高、接替有序。安排3000万元，专项用于重大项目前期工作，重点做好关天规划中明确的重大基础设施、微型车发动机、铁道电器研发生产、水源工程等28个项目的前期工作，积极创造条件，确保一批成熟项目尽早开工建设。

　　加大招商引资工作力度。坚持招商引资“一把手工程”不动摇，立足招大商选好商，强化工作措施，促进重大项目落地。围绕大企业大集团产业链延伸、文化旅游产业振兴等重大项目，组织策划并参与专项招商活动，着力引进一批大项目。充分发挥上海办、深圳办等驻外机构优势，建立长效稳定的招商机制和渠道。加大对签约项目的跟踪落实力度，提高合同履约率、资金到位率和项目开工率，确保全年实现合同引资500亿元，到位资金200亿元以上。

　　（五）加快特大城市建设，打造最佳宜居城市

　　坚持走新型城市化道路，以建设生态宜居城市为目标，扩张城市规模，完善服务功能，加快要素聚集，全面提升特大城市规划建设和管理水平。

　　超前建设重大基础设施。按照“超前规划、系统布局、加快建设”的原则，以重大基础设施建设为突破口，推动特大城市建设全面提速。加快西宝高速改扩建、宝平高速、西宝快速铁路客运专线、滨水大道等项目建设，力争开工建设宝汉高速、合凤高速宝鸡段、连霍线宝鸡过境段改线、麟北运煤专线、宝兰铁路客运专线和宝鸡南客站，积极做好县县通高速、渭河南铁路枢纽专线、城市轻轨、宝鸡支线机场等前期工作，着力构建现代化城市交通体系。抓紧建设宝鸡环城高速、秦岭北麓旅游观光大道和310国道市区过境段改线工程前期工作，完善城市环形道路骨架，带动蟠龙新区和秦岭北麓开发建设。加快陈仓区市政基础设施建设步伐，加速市区与陈仓区融合。

　　加快蟠龙新区开发。按照宝鸡特大城市蟠龙组团的规划定位，坚持综合开发、集约资源的原则，把新区建设成为基础设施最好、体制机制最活、承载能力最强的现代化新城区。成立新区开发建设管理机构，切实做好园区开发策划，制定产业发展详规。开工建设上塬道路、东西大道、新区引水等工程，加快推进蟠龙新区基础设施建设。

　　完善城市公共服务功能。瞄准特大城市目标定位，不断加强公共服务设施建设，最大限度地满足城市经济社会发展需要。加快宝鸡南客站片区、陈仓物流园区的基础设施配套建设，抓好高新东区、代家湾等片区学校、医院、市场、社区等公共设施建设，方便群众生产生活。前瞻性做好城市地下工程的系统规划，一次性解决新建城区地下水电、气暖、通讯等问题。加大中山路、群众路、清姜路沿线基础设施改造力度，抓好南关路、敦仁堡等片区旧城和城中村改造，实施金陵新村等旧住宅小区设施改造，提升老城区环境品位。积极实施“气化宝鸡”工程，扩大天然气用户覆盖面。实施集中供热工程，新建一批供热管网和换热站。抓好石头河引水、引红济石等重大水利工程建设，提高城市供水安全保障能力。以创建全国和谐社区示范单位为抓手，发展医疗保健、家政服务、养老托幼和食品配送等服务，提高社区便民服务水平。

　　着力改善生态环境。抓好金陵河上游市区段综合治理，完成石鼓山坝桥结合工程，开工建设行政中心段橡胶坝项目，改善城市水生态环境。继续实施渭河、小韦河和千河流域综合治理，建成一批国家水利风景区。持续推进绿色宝鸡建设，重点抓好“四旁”绿化和重点区域绿化，建成开放代家湾北坡森林公园，完成南山4个坡地公园建设，打造西部精品园林城市。努力创建全省首个国家生态示范市，确保顺利通过国家环保模范城市复检验收。

　　全面提升管理水平。严格城市规划管理，推进城市管理精细化、科学化、现代化。加快高新东区数字化城管系统建设，逐步实现城市管理无缝隙、不间断、全覆盖。加强城市管理综合执法，深入开展环境综合整治，改善市容市貌。通过新建停车场、开发利用地下空间、架设人行天桥、整治市区交通秩序、引导人流物流向新城区分流等综合措施，提高道路利用率，着力解决老城区交通拥堵和停车难等问题。切实规范和提升房地产规划建设管理水平，推行标准化物业管理，提高业主居住质量。加强环卫保洁工作，营造舒适优美的生活环境。

　　（六）推动园区超常规发展，打造经济增长新引擎

　　大力实施园区带动战略，创新体制机制，优化资源配置，推进产业聚集，把“两区多园”建成引领全市经济发展的示范区。

　　加快高新区率先发展。以建设一流创新型园区为目标，推进科技新城建设，成立宝鸡高新科技公司，抓好科技创新园和汽车零部件创业孵化基地建设，促进高新区由投资驱动向创新推动转变。完善高新区管理体制，对周边乡镇的人、财、物实行托管，支持高新区高速成长。抓好高新大道三期建设，完成高新二期渭河南堤绿化和滨河路建设，提升高新区承载服务功能。年内高新区生产总值力争突破300亿元。

　　支持蔡家坡开发区跨越发展。加快建设国家重型汽车及零部件特色产业基地，抓好陕汽30万辆微型车、汉德车桥扩建技改、通力专用汽车等项目建设，确保6月底前微型车下线。突出汽车及零部件产业主导地位，实施一批园区配套项目，增强产业和基础设施承载能力，推动重大汽车工业项目向蔡家坡集中。改革蔡家坡管委会运行体制，托管周边乡镇，实行独立运行，搞活分配机制，加快蔡家坡工业化、城镇化进程。

　　推动县域工业园突破发展。把工业园区作为县域工业化的重要载体，围绕为大工业配套、发展农产品加工业、依托优势资源延伸产业链、发展科技创新型企业等重点，突出特色，错位发展，把园区的产业做专做精、做大做强。发挥5000万元园区专项资金作用，努力在基础设施建设、做实投资公司和引进企业入园上实现新的突破。全面落实财政扶持、用地保障、融资担保等各项政策措施，切实解决中小企业融资难、用地难等问题，培育更多的非公企业和个体工商户，构建以非公经济为主体的县域经济发展格局。

　　（七）做大做强文化旅游产业，提升宝鸡对外影响力

　　充分发挥资源优势，打响文化旅游品牌，推进文化与旅游的融合，把文化旅游产业打造成新的主导产业。

　　大力弘扬宝鸡特色文化。深度挖掘以姜炎文化、周秦文化为主的历史文化，以民间艺术、西府民俗为主的传统文化，整合文化资源，积极发展广告艺术、文化创意、网络动漫等新型文化产业。落实和完善文化创新的各项政策，吸引民间资本进入文化市场。加快广电中心建设，新建宝鸡图书馆，做好宝鸡大剧院、市群众文化活动中心等项目的前期工作。加强基层文化设施建设，新建一批县级文化资源共享中心、乡镇综合文化站、农家书屋。举办“九成宫”杯全国书画大赛和宝鸡民间文化艺术节，开展文化惠民演出和送戏曲、送电影下乡活动，支持公园、广场等群众性文化娱乐活动，满足广大群众文化需求。

　　加快实施重点旅游项目。以打造国内一流旅游目的地为目标，优化旅游业布局，开发精品旅游项目，加快文化旅游产业发展。建设周秦文化产业示范区，实施中华礼乐城二期、石鼓山古玩城等重点项目，力争国庆节前青铜器博物院建成开放，形成都市旅游热点。加快法门寺文化景区二期工程建设，创建并申报国家 5A级景区。加快太白山景区开发建设，提升景区管理水平。积极发展关中民俗风情和历史古迹游、岭南山水休闲游、关山草原风光游，抓好西岐民俗园、六营民艺村等乡村旅游示范点，做火做旺旅游市场。

　　提高旅游业综合服务水平。积极参加各类旅游博览会、展览会，加强区域交流与合作，提高宝鸡旅游的知名度和影响力。推行标准化管理，推动一批景区、旅行社、旅游宾馆上档升级。加大从业人员的培训力度，不断提升旅游管理水平和服务质量。切实抓好旅游景点配套设施建设，建成一批游客服务中心，开通各大景点间的旅游班车，绿化美化旅游干线，完善服务功能。力争年内接待游客 1500万人次，实现旅游综合收入 100亿元。

　　打造区域商贸物流中心。以大物流、大交通为目标，构建物流网络，搭建公共信息平台，形成快进快出的现代化流通体系，把我市建成承东启西、沟通南北的物流集散地。加快陈仓物流园区总体规划编制步伐，进一步理顺园区管理体制，整合现有物流资源，建设保税物流场所，带动物流、资金流向宝鸡汇集。支持华誉物流、西部物流等龙头企业发展，促进装备制造企业与物流企业联动，降低社会物流运营成本。优化城市商业布局，加快高新东区商业网点规划建设，推进石坝河、清姜、福谭、上马营、代家湾、陈仓等6个区域性商业中心建设。大力实施“万村千乡”、“双百市场”工程和“新网工程”，积极开展“农超对接”试点，推动城市商业向农村延伸。继续落实家电和汽车以旧换新政策，推进家电、农机、汽车、摩托车下乡。加强城乡市场价格监管，促进城乡消费。

　　加快发展现代服务业。把现代服务业作为结构调整的战略重点，大力发展工业设计、技术研发、信息咨询、商务会展等生产性服务业，积极发展中介、创意、服务外包等新兴服务业。加大金融生态环境和信用环境建设力度，积极引进民生、中信、招商等股份制银行和证券、保险机构来宝设立网点，培育壮大金融服务业。选择一批优势企业，与深圳、上海创投公司或基金公司开展合作，拓宽融资渠道。做实做强市投资（集团）公司，力争发行债券15亿元。

（八）深入推进改革开放，增强加快发展的活力

　　以更大的气魄、更大的力度，扎实推进重点领域和关键环节的改革，形成更具活力、更加开放的体制机制，为宝鸡新一轮加快发展注入强大活力。

　　继续深化国企改革。以股权多元化为目标，积极引进战略投资者，支持鼓励大企业进行战略重组，全面启动商贸流通企业民营化改革，激发国有企业整体活力。组建麟北煤田联合投资体，完成西凤酒集团增资扩股，力争红旗民爆年内上市融资。支持引导困难企业改制重组，加快实施陕棉九厂政策性破产，完成宝鸡酒精厂、双菱化工集团等企业的政策性破产重组。

　　加大农村改革力度。深化农村综合改革，不断增强农业、农村经济发展活力。积极组建村镇银行和小额贷款公司，鼓励商业性保险机构开发“三农”保险市场，搞活农村金融。完善农业经营体制，积极稳妥地推进农村土地承包经营权流转，促进农业集约化经营。推行水权制度改革，促进水资源高效利用。加快推进集体林权制度改革，太白、渭滨等5个县区有效实施配套改革，其他7个县全面完成主体改革任务。

　　加快公共服务领域改革。完成市县政府机构改革任务，推进乡镇机构改革。加速文化体制改革步伐，全面完成市直单位文化体制改革任务。以国家医药卫生体制改革试点为契机，积极推进城乡基层医疗机构药品“三统一”和公立医院改革试点，健全农村卫生服务网络和城市社区卫生服务体系，让广大群众在医改中得到实惠。继续推进教育改革，逐步推行免费中等职业教育，深化中小学教师职称制度改革，推动教育事业全面发展。

　　（九）着力改善民生，促进社会和谐

　　把保障和改善民生作为政府工作的出发点和落脚点，继续加大投入力度，不断改善民生，努力提高全市人民的幸福感，促进社会和谐。

　　提高社会保障水平。进一步提高新型农村社会养老保险参保率，全面启动城镇居民基本养老保险工作，实现城乡居民养老保险全覆盖。加快城镇职工医疗保险市级统筹步伐，切实抓好城镇居民医疗保险扩面工作。加强新型农村合作医疗运行管理，全面推行门诊统筹报销制度。加大城乡低保、医疗救助以及特定民政对象救助力度，完善规范化、精细化、动态化管理制度，对城乡低保边缘群体和低收入家庭实施延伸救助，建立“多层次、全覆盖、广普惠”的社会救助体系。加快经济适用住房和廉租房建设，切实解决中低收入家庭的住房保障问题。创建全国残疾人工作示范城市，努力解决残疾人康复、教育、就业和生活保障等方面的突出困难，为残疾人办好十件实事。实施社会事业公共服务平台项目，12个县区市民中心“五•一”前全部投用。完善社会保障网络体系建设，信息网络延伸到每个社区和每个行政村。加快城镇居民住房、学校和医疗机构重建步伐，8月底前全面完成灾后重建任务。

　　积极推进创业就业。以创建全国创业型城市为契机，整合政策和资源，在高新区设立大学生创业孵化中心，在每个工业园区建立创业孵化基地，在每个乡镇建立一个农业科技示范园，在城市街道建设一批创业示范市场，为各类人员搭建创业平台。建立市、县区创业服务中心，面向所有创业者开展免费创业培训，办好创业信息服务平台，增加创业计划和项目，将创业项目库连接到每个社区和行政村。鼓励各类人员自谋职业、自主创业，并将其纳入灵活就业人员社保补贴范围。加大对创业者的信贷支持，力争全年完成小额担保贷款 2亿元以上。全面落实税费减免等扶持政策，大力实施“人人技能工程”，打响“宝鸡技工”品牌，促进各类人员充分就业。进一步开发农村公益性就业岗位，实施对“零就业”家庭、低保家庭等困难群体的援助就业。年内实现城镇新增就业 4.6万人、下岗失业人员再就业 1.8万人，转移农村劳动力 100万人。

　　统筹发展社会事业。加快教育布局调整，抓好义务教育提升工程和中小学校舍安全工程，实施市区中小学布局规划，促进城乡义务教育均衡发展。加强民办教育规范化管理，加快职业教育发展，努力将宝鸡职业技术学院创建成全省示范性高职院校。支持宝鸡文理学院硕士学位授权单位立项建设，提升高等教育办学层次。大力开展全民健康教育，完成“十一五”重点地方病防治规划目标，加强重点传染病防治工作。开展食品药品安全专项整治行动，确保群众饮食、用药安全。加快扶风、岐山、太白、陇县等国家卫生县城创建步伐，力争年内有 1—2个县创建成功。扎实开展第六次人口普查，做好人口计划生育工作，建立以县为主、统筹解决人口问题的新机制，稳定低生育水平。积极应对人口老龄化趋势，加大对老龄事业的投入力度，加快社会化养老服务中心建设，完善养老服务体系。保障妇女儿童合法权益，加强青少年思想道德建设，促进妇女儿童事业全面发展。抓好城乡体育设施建设，积极组团参加全省第14届运动会，大力开展群众性体育运动。做好档案信息化建设，抓好第二轮续修地方志工作。

　　全力维护社会和谐稳定。加强社会治安综合治理，严厉打击各类刑事犯罪活动，加大社会治安重点地区排查整治力度，完善网格化治安巡防体系，深化全省最安全城市创建活动，提高人民群众的安全感。强化维稳工作责任制，健全社会稳定风险评估机制。加大社会矛盾纠纷调处化解力度，引导群众以理性合法方式表达利益诉求，完善领导干部接访、下访、包案制度，努力从源头上解决信访突出问题。认真开展“安全生产年”活动，加大道路交通、非煤矿山、危险化学品、人员密集区等重点行业和部位的安全监管力度，及时整改安全生产隐患，有效遏制重特大事故发生。加快应急体系建设，提高减灾救灾能力。加快少数民族聚居地方经济社会事业发展，发挥宗教界在构建和谐社会中的积极作用。抓好国防教育、人民防空、民兵预备役建设，巩固全国双拥模范城建设成果。

全面加强政府自身建设

　　各位代表，市委十届七次全会明确提出了今年工作的思路和目标，全面完成今年各项任务，市政府肩负着重大责任。我们要切实加强政府自身建设和干部队伍建设，引导广大干部把学习作为必备的生活习惯，把创新作为基本的思维方式，把吃苦作为良好的工作作风，努力建设群众真心拥护、人民真正满意的服务型政府。

　　深入解放思想。面对新的发展形势、发展任务、发展要求，全体政府工作人员必须加强学习，掌握各自工作的理论和实践前沿，增强适应新生事物的能力。要大胆解放思想、更新发展理念，着力转变不适应、不符合科学发展观的思想认识，破除因循守旧观念，增强开放合作意识、改革创新意识、开拓进取意识，不断消除影响发展的体制机制性障碍，用创新的办法破解发展难题。要敢于同沿海发达地区比，同周边先进地区比，在横向比较中找差距、定目标，不断增强忧患意识和紧迫感，以更高的标准要求自己，把宝鸡的改革开放和现代化建设不断推向前进。

　　坚持科学决策。实施重大事项科学决策制度，坚持调查研究、专家咨询、公众参与、社会公示、集体讨论等决策程序，充分发挥市决策咨询委员会的作用，努力提高领导科学发展的能力和水平。不断创新决策方式，有效运用规划等手段管理经济社会事务，规范部门决策行为，对涉及经济社会发展的重大事项都要进行规划，所有中长期发展规划都聘请国际国内一流的规划设计单位参与，达到国内一流水平，从发展源头上实现与先进发达地区的接轨。

　　推进依法行政。全面推进法治政府建设，完善行政执法责任制，推行行政处罚自由裁量权基准制度，深入开展创建依法行政示范县区活动，努力做到依法行政、程序正当、高效便民、权责统一。加快电子政务建设，加大政务信息公开力度，推进部门和企事业单位办事公开。进一步发挥市行政服务中心的功能与作用，应纳入事项全部驻厅办理。继续加强依法治市（县）工作，全面推行村民监督委员会制度，加快村务公开民主管理试点，做好第四届城市社区居委会换届工作。自觉接受人大及其常委会的工作监督和法律监督、政协的民主监督和社会公众监督，确保行政权力正确行使。

　　狠抓工作落实。按照关天规划和2020年全面实现小康社会的目标要求，建立经济社会综合指标和工业发展、大城市建设、农民增收、第三产业、城乡统筹五个专项指标体系，逐年量化分解目标和任务，建立科学考评体系。大力弘扬密切联系群众之风、求真务实之风，坚持领导干部工作联系点制度、政府有关部门公开服务热线电话制度和网上信箱制度，积极倾听群众呼声和社情民意，切实解决基层群众的实际困难和问题。深入开展机关纪律作风整顿，着力解决干部作风方面的突出问题。加强机关行政效能建设，健全和落实首问负责制、限时办结制、责任追究制，严肃查处政令不畅、作风漂浮等问题，营造一门心思谋发展的浓厚氛围。

　　加强廉政建设。认真落实廉政建设责任制，严格工程招标、土地出让、产权交易、资源使用、重大项目投资、政府采购等制度建设，从源头上杜绝违法乱纪行为发生。深入开展工程建设领域突出问题专项治理，严禁领导干部违规插手干预工程建设项目。深化“小金库”专项治理工作，并向社会团体和国有企业延伸。积极解决群众反映强烈的突出问题，严肃处理吃拿卡要等破坏投资环境的行为。加强财政预算执行审计，压缩行政会议、接待、办公等方面的经费支出。进一步加强公车管理，控制公车总量增长。加强廉政文化建设，增强公务人员廉政意识和抗腐蚀能力，形成风清气正、干净干事的良好局面。

　　各位代表，我们面临的机遇千载难逢，挑战前所未有。实现宝鸡率先发展的宏伟目标是我们的光荣使命，让我们在市委的正确领导下，凝聚全市人民的智慧和力量，万众一心谋发展，群策群力绘蓝图，为夺取经济社会发展新成就、谱写全市人民幸福生活新篇章而奋斗！
